# Linearity Enhancement in Magnetostrictive Sensors Based on Substructure with Tunable Poisson’s Ratio

**DOI:** 10.3390/s26123792

**Published:** 2026-06-14

**Authors:** Shuairan Xu, Xu Zhang, Jianyu Song, Yisong Tan

**Affiliations:** School of Mechanical Engineering, Northeast Electric Power University, Jilin 132012, China; 2202301091@neepu.edu.cn (S.X.); 33520250157483@xmu.edu.cn (X.Z.); songjianyu2002@163.com (J.S.)

**Keywords:** magnetostrictive sensor, linearity optimization, tunable Poisson’s ratio, chiral auxetic honeycomb, bearing press-fit force

## Abstract

Magnetostrictive sensors based on the inverse magnetostrictive effect offer the advantages of wireless passive operation and structural simplicity; however, achieving both high sensitivity and superior linearity remains a persistent challenge. This study presents a magnetostrictive pressure sensor incorporating a tunable Poisson’s ratio (TPR) chiral auxetic honeycomb substructure, designed to linearize the stress response of the sensing material. A theoretical model linking substructure design parameters to sensor output linearity was derived and validated through finite element simulations. The fabricated substructure exhibited a stable negative Poisson’s ratio (−1.278 to −1.213) within its elastic regime and a highly linear axial-to-transverse strain relationship (x = 1.214y + 0.113). The sensor achieved a calibration linearity of R^2^ = 0.99745, a continuous linear force response up to 118.7 N while the corresponding voltage variation reached 10.75 mV, and a maximum hysteresis error of 5.495% over eight loading cycles. Bearing press-fit force monitoring experiments confirmed practical viability under industrial conditions, with R^2^ exceeding at least 0.995 for dry assembly between multiple bearing types and maintaining R^2^ > 0.994 under lubricated conditions. The proposed TPR substructure approach establishes a reference framework for linearity enhancement in inverse magnetostrictive force sensors.

## 1. Introduction

The magnetostrictive sensors overcome the limitations of conventional sensors that require power supplies, which necessitate complex wiring arrangements. As a result, they have found extensive applications in industrial manufacturing [1,2], biomedical engineering [3,4,5,6], contactless sensing [7,8,9], and intelligent robotics [10,11]. Among these, magnetostrictive sensors fabricated from isotropic magnetostrictive materials, particularly iron-based amorphous alloys, have attracted significant research interest and widespread adoption in these fields. This is attributed to their structurally stable and reliable performance, excellent thermal adaptability, and low cost.

The direct magnetostrictive effect refers to the dimensional change in magnetostrictive materials along the magnetic induction direction when subjected to an external magnetic field during magnetization. Conversely, the inverse magnetostrictive effect (also known as the piezomagnetic effect or Villari effect) describes the alteration of the material’s magnetic field resulting from the reorientation of magnetic domains when mechanical stress is applied. Conventional magnetostrictive sensors based on these two effects typically exhibit limited performance in terms of sensitivity and linearity. Recent studies have developed various approaches to optimize magnetostrictive sensor performance, and the primary methods include:

1. Optimization of sensing element geometry and dimensions [12,13], which primarily utilize the direct effect and require advanced fabrication techniques.
2. Adjustment of bias magnetic field and optimization of detection system parameters [14,15].
3. Coating or adhering a composite layer of non-sensitive material on the sensing material [16,17].
4. Modification of substructure materials or design of specially structured substructures [18,19].

Among these approaches, substructure design has demonstrated particularly promising results in current research and is expected to become a key direction for future performance enhancement of magnetostrictive sensors.

Extensive studies have been conducted on the direct improvement of sensitivity and its trade-off with measurement range in magnetostrictive sensors, establishing relatively reliable theoretical frameworks. William S. Skinner [15] and Paula G. Saiz [20] investigated the sensitivity enhancement principles of direct magnetostrictive effect sensors by modifying the geometric configurations of magnetostrictive materials. Daniel Matatagui [21] examined the sensitivity of amorphous magnetostrictive sensors using a customized detection system, specifically employing Helmholtz coils to analyze the relationship between frequency shift and deposited mass loading. Chuan Xie [22] proposed an improved detection system for magnetostrictive sensors based on the maximum induced voltage method. Moyue Cong [18] developed a wireless passive magnetostrictive strain sensor with enhanced sensitivity, where the optimization effect was achieved through a specially designed negative Poisson’s ratio substructure.

However, current research on magnetostrictive sensor performance predominantly focuses on singular sensitivity enhancement or comprehensive optimization of both sensitivity and measurement range. Notably, sensors designed through substructure optimization approaches typically exhibit nonlinear output curves or piecewise linear characteristics within their operational range. This behavior significantly compromises the accurate quantitative calibration between output signals and applied loads.

To address the aforementioned challenges, this study focuses on designing a variable Poisson’s ratio substructure for enhancing the linearity of magnetostrictive pressure sensors. The research objectives are threefold:

1. To achieve a structure with excellent mechanical properties that meets the needs of magnetostrictive sensors by optimizing the substructure design.
2. To optimize the design of the substructure and sensor, and complete the development of a magnetostrictive sensor with enhanced linearity compared to existing research.
3. To conduct practical performance experiments on the sensor to demonstrate the realization and significance of the linearity optimization.

This work aims to establish a reference for linearity improvement in magnetostrictive sensors under force detection conditions, achieving comprehensive improvement of mechanical properties and sensing performance through TPR structural innovation design. The experimental validation will include overall performance evaluation of the proposed sensor design, obtaining the enhancement of sensing performance indicators.

## 2. Material and Methods

### 2.1. Sensing Material

Magnetostrictive sensing materials can be primarily categorized into wire-type and bulk-type materials. Bulk magnetostrictive materials, including Fe-Ga alloys [1] and Terfenol-D [23], exhibit rapid signal response characteristics. However, their applications are limited due to inherent brittleness and high manufacturing costs. In contrast, wire-type magnetostrictive materials demonstrate superior mechanical properties and lower production costs, making them more widely applicable. Typical examples include isotropic iron-based amorphous alloys such as Metglas2826MB [24,25] and Metglas2605SA1 [26,27].

Therefore, Metglas2826MB, a magnetostrictive material composed of 40% Fe, 38% Ni, 4% Mo, and 18% B, was selected as the sensing material for the sensor in this study. This iron-based amorphous alloy ribbon can be processed to a thickness of only 28 μm, which exerts a negligible impact on the mechanical characteristics of the substructure. Furthermore, the sensing elements can be directly tailored from the bulk material according to the dimensional specifications of the substructure, ensuring a highly convenient integration process within the magnetostrictive sensor. Additionally, the material’s high magnetic permeability and excellent isotropic magnetostrictive properties are highly compatible with the requirements of the proposed sensor.

With a modest magnetostrictive coefficient of 12 ppm, this material requires innovative substructure design strategies to enhance sensitivity. Existing research has demonstrated that a deliberately engineered substructure capable of generating transverse strain with the same polarity as the principal strain direction can significantly amplify the output signal. This principle underlies the sensitivity enhancement mechanism for inverse magnetostrictive effect sensors in current studies. By designing a substructure with tunable mechanical properties such as Poisson’s ratio, the substructure characteristics can be precisely matched with the sensing features of magnetostrictive materials. This approach enables linearization of the output signal in magnetostrictive pressure sensors, ultimately yielding detection results with superior calibration performance.

### 2.2. Substructure of the Proposed Sensor

**Figure 1:**
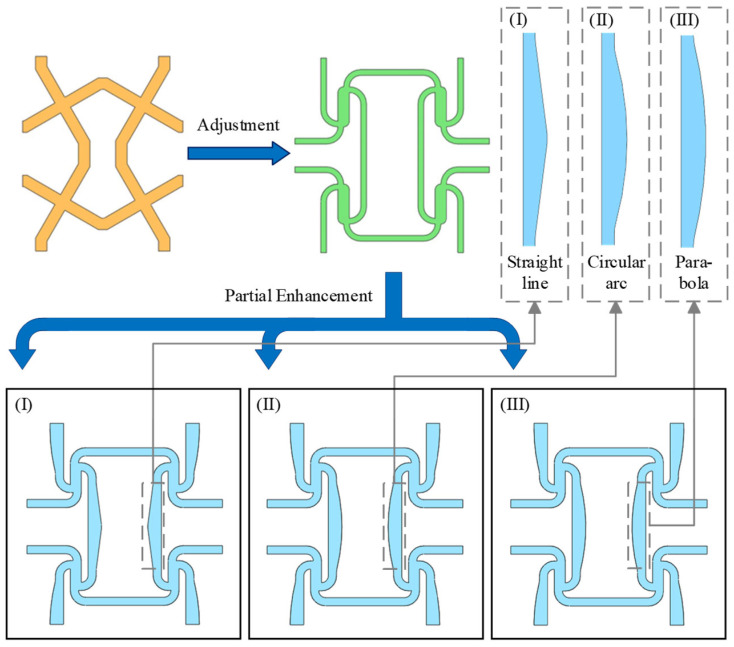
Optimization process and design features of the honeycomb unit.

To achieve substantial strain under pressure loading while maintaining a tunable quantitative relationship between Poisson’s ratio and strain development, careful selection of the honeycomb unit configuration is essential to obtain appropriate deformation modes. This study employs a chiral auxetic structure configuration, specifically designed to exhibit tunable Poisson’s ratio (TPR) characteristics with large absolute values. The honeycomb unit features axisymmetric geometry, with the detailed optimization process and design features shown in Figure 1.

In current research, magnetostrictive pressure sensors with high sensitivity typically exhibit convex output curves or piecewise linear responses with stable slopes across their measurement ranges. To address this characteristic, the honeycomb design parameters of the substructure must be adjusted to modify the Poisson’s ratio profile into a continuously stable curve. This optimization enables the magnetostrictive sensor to produce output signals that maintain high linear correlation with the applied pressure.

**Figure 2:**
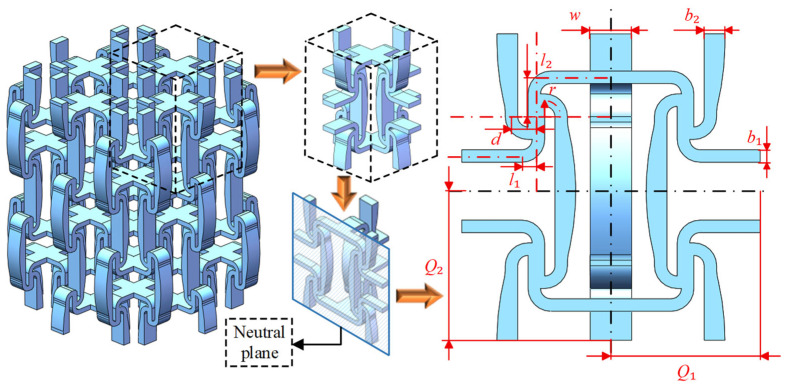
Main design parameters of tunable Poisson’s ratio substructure.

The design parameters of the chiral substructure are illustrated in Figure 2. To avoid misalignment or intersection during geometric splicing, design parameters should meet the constraints as in Equation (1):

(1) $$\left\{\begin{array}{l} Q_{1}>w+2(d+b_{2})-b_{1}\\ Q_{2}>2l_{2}+b_{1}\\ Q_{1}=Q_{2}=Q\\ d\geq r\\ l_{1}\geq 0\\ l_{2}=2r+\frac{b_{1}}{2}\\ b_{1}\leq b_{2}\leq 2b_{1} \end{array}\right.$$

where $Q_{1}$, $Q_{2}$ represents the horizontal length and vertical height of the quarter-cell, both of which are set to equal values. $b_{2}$ represents the enhanced structure width correlated with the functional law of the enhanced structure in the unit cell. $l_{1}$, $l_{2}$, $b_{1}$, d, r, and w represent conventional unit design parameters.

### 2.3. Model and Theoretical Analysis

**Figure 3:**
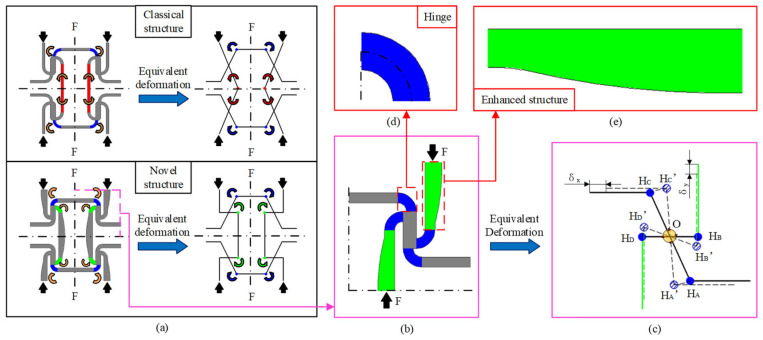
(**a**) Comparison of deformation patterns between two chiral honeycomb structures. (**b**) Representative unit cell of novel structures. (**c**) Quarter-cell deformation model. (**d**) Circular arc hinge structure. (**e**) Enhanced structure.

A simplified computational model was established for the honeycomb unit of the substructure. As shown in Figure 3, under compressive strain, the solid lines represent the initial positions of the honeycomb structure before compression, while the dashed lines indicate the deformed positions after compression. Here, $\delta_{x}$ and $\delta_{y}$ denote the unilateral deformation values in the compressive direction and lateral direction, respectively.

Based on honeycomb design parameters, we can obtain:

(2) $$\left\{\begin{array}{l} H_{A}H_{C}=2{\mathrm{OH}}_{A}=2d\\ H_{B}H_{D}=2{\mathrm{OH}}_{B}=2\sqrt{l_{1}^{2}+l_{2}^{2}} \end{array}\right.$$

Assuming that only the inclined members CD and AB undergo rotation, the hinge points $H_{A}\sim H_{D}$ are approximately ideal hinges; it can be inferred that the displacement projections of each symmetric boundary point along the component axis direction are $\delta_{x}$ and $\delta_{y}$, both of which can be represented by design parameters.

The nodal flexibility of circular arc components can be represented as the following matrix:

(3) $$S_{1}^{\left(i\right)}=\left[\begin{array}{ccc} a_{11}^{\left(i\right)} & a_{12}^{\left(i\right)} & a_{13}^{\left(i\right)}\\ a_{12}^{\left(i\right)} & a_{22}^{\left(i\right)} & a_{23}^{\left(i\right)}\\ a_{13}^{\left(i\right)} & a_{23}^{\left(i\right)} & a_{33}^{\left(i\right)} \end{array}\right]$$

For the enhanced components, assuming that there is a functional relationship $b_{i}=f(l_{i})$ between the thickness of the component and the distance to the symmetry axis of the unit, its nodal flexibility can be represented as the following matrix:

(4) $$S_{2}^{\left(i\right)}=\left[\begin{array}{ccc} c_{11}^{\left(i\right)} & 0 & 0\\ 0 & 0 & c_{23}^{\left(i\right)}\\ 0 & c_{23}^{\left(i\right)} & c_{33}^{\left(i\right)} \end{array}\right]$$

The compliance constants and coupling terms of circular arc components are calculated using the following formulas:

(5) $$\left\{\begin{array}{l} a_{11}^{\left(i\right)}=\frac{\pi r}{4E_{m}wb_{1}}+\frac{3r^{3}}{E_{m}wb_{1}^{3}}\left(3\pi -8\right)\\ a_{12}^{\left(i\right)}=-\frac{r}{2E_{m}wb_{1}}+\frac{6r^{3}}{E_{m}wb_{1}^{3}}\\ a_{13}^{\left(i\right)}=\frac{6r^{2}}{E_{m}wb_{1}^{3}}\left(2-\pi \right)\\ a_{22}^{\left(i\right)}=\pi \left(\frac{r}{4E_{m}wb_{1}}+\frac{3r^{3}}{E_{m}wb_{1}^{3}}\right)\\ a_{23}^{\left(i\right)}=-\frac{6r^{2}}{E_{m}wb_{1}^{3}}\\ a_{33}^{\left(i\right)}=\frac{6\pi r}{E_{m}wb_{1}^{3}} \end{array}\right.$$

where $E_{m}$ represents the Young’s modulus of material.

The compliance constants and coupling terms of enhanced components are calculated using the following formulas:

(6) $$\left\{\begin{array}{l} c_{11}^{\left(i\right)}=\int_{0}^{\frac{L_{i}}{2}}\frac{1}{E_{m}wf(l_{i})}dl_{i}\\ c_{23}^{\left(i\right)}=\int_{0}^{\frac{L_{i}}{2}}\frac{6\left(L_{i}-2l_{i}\right)}{E_{m}w{\left[f(l_{i})\right]}^{3}}dl_{i}\\ c_{33}^{\left(i\right)}=\int_{0}^{\frac{L_{i}}{2}}\frac{12}{E_{m}w{\left[f(l_{i})\right]}^{3}}dl_{i} \end{array}\right.$$

where $L_{i}$ in the upper limit of integration represents the total length of enhanced components.

The transformation matrix during the process of assembling nodes into component units is:

(7) $$R_{i}=\left[\begin{array}{ccc} -\cos\phi_{i} & -\sin\phi_{i} & 0\\ \sin\phi_{i} & -\cos\phi_{i} & 0\\ r(1-\cos\phi_{i}) & r\sin\phi_{i} & -1 \end{array}\right]$$

where $\phi_{i}$ is the central angle corresponding to each circular arc component, and it is $\frac{\pi }{2}$ in this paper.

The local flexibility matrix of the component obtained through flexibility decoupling is:

(8) $$S_{i}=\left[\begin{array}{cc} S^{\left(i\right)} & S^{\left(i\right)}R_{i}^{T}\\ R_{i}S^{\left(i\right)} & S^{\left(i\right)} \end{array}\right]$$

Furthermore, the transformation matrix $T_{i}$ during the assembly of members into a honeycomb unit is expressed as:

(9) $$\left\{\begin{array}{l} T_{i}=\left[\begin{array}{cc} t_{i} & 0\\ 0 & t_{i} \end{array}\right]\\ t_{i}=\left[\begin{array}{ccc} \cos\theta_{i} & \sin\theta_{i} & 0\\ -\sin\theta_{i} & \cos\theta_{i} & 0\\ 0 & 0 & 1 \end{array}\right] \end{array}\right.$$

where $\theta_{i}$ represents the angle between the axis of each component and the direction of the pressure applied to the honeycomb.

The equivalent transformation matrix for component stiffness is:

(10) $$K_{r}^{i}=T_{i}^{-1}S_{i}^{-1}T_{i}$$

The global stiffness matrix of the honeycomb unit can then be expressed as:

(11) $$K_{g}=\left[\begin{array}{ccccc} K_{r11}^{\left(1\right)} & K_{r12}^{\left(1\right)} & 0 & \ldots & 0\\ K_{r21}^{\left(1\right)} & K_{r11}^{\left(2\right)}+K_{r22}^{\left(1\right)} & K_{r12}^{\left(2\right)} & \ddots & \vdots \\ 0 & K_{r21}^{\left(2\right)} & \ddots & K_{r12}^{\left(n-1\right)} & 0\\ \vdots & \ddots & K_{r21}^{\left(n-1\right)} & K_{r11}^{\left(n\right)}+K_{r22}^{\left(n-1\right)} & K_{r12}^{\left(n\right)}\\ 0 & \ldots & 0 & K_{r21}^{\left(n\right)} & K_{r22}^{\left(n\right)} \end{array}\right]$$

where n denotes the number of structural components in the honeycomb unit.

According to the generalized Hooke’s law of elasticity, the force acting on a honeycomb is related to its stiffness matrix and the strain as follows:

(12) $$F=K_{g}\delta$$

where F is the stress vector and δ is the strain vector.

Consequently, the theoretical relationship between Poisson’s ratio and honeycomb design parameters is obtained as follows:

(13) $$\nu_{xy}=-\frac{\varepsilon_{x}}{\varepsilon_{y}}=-\frac{\delta_{x}}{\delta_{y}}=-\frac{{\left(5l_{1}+b_{1}\right)}^{2}\left(b_{1}+2\pi r+1\right)\left(2b_{1}+2r+d-l_{1}\right)}{{\left(b_{1}+l_{1}\right)}^{2}\left(b_{1}+l_{1}+4\right)\left(8d+18\pi r\right)}$$

Since the honeycomb unit of the substructure has identical structural composition in both the *x* and *z* directions, it follows that:

(14) $$\nu_{zy}=\nu_{xy}=\nu$$

Through equivalent calculation, the equivalent Young’s modulus of the three-dimensional honeycomb is:

(15) $$E_{3D}=\frac{\sigma_{3D}}{\varepsilon_{y}}=\frac{E_{m}\left(2Q-w\right)wb_{1}^{3}}{12\pi r^{3}Q^{2}}$$

The total stressed cross-sectional area of the sensor is:

(16) $$A=m^{2}Q^{2}$$

where m is the periodic array number of honeycomb cells in the substructure.

The axial strain generated by the sensor under the action of y-axis pressure is:

(17) $$\varepsilon_{y}=\frac{F_{y}}{E_{3D}A}$$

The transverse strains in the *x* and *z* directions generated by the tunable Poisson’s ratio substructure are:

(18) $$\varepsilon_{x}=\varepsilon_{z}=-\nu \varepsilon_{y}$$

Based on the ideal elastic model in elasticity theory, the stress–strain expression of the substructure can be obtained as follows:

(19) $$\sigma =E_{3D}\varepsilon_{y}\left(1-2\nu \right)=\frac{F_{y}}{A}\left(1-2\nu \right)$$

For a magnetostrictive sensor employing 2826MB as the sensing material, the induced voltage in the detection coil can be expressed as [28]:

(20) $$\overset{\cdot }{U_{2}}=j\omega \overset{\cdot }{I_{1}}\frac{\mu_{0}^{2}NS_{2}}{\mu_{1}}\left(1+\frac{M_{s}^{2}}{2K_{u}-3\lambda_{s}\sigma }\right)$$

where j, ω, $\dot{I_{1}}$, $\mu_{0}$, N, $S_{2}$, and $\mu_{1}\;$are parameters of the detection system. $M_{s}$, $K_{u}$, and $\lambda_{s}\;$are magnetic parameters of the sensing material. σ is stress, and all parameters except stress σ are constants.

Substituting Equation (19) into Equation (20) yields:

(21) $$\overset{\cdot }{U_{2}}=j\omega \overset{\cdot }{I_{1}}\frac{\mu_{0}^{2}nS_{2}}{\mu_{1}}\left(1+\frac{M_{s}^{2}A}{2K_{u}A-3\lambda_{s}F_{y}\left(1-2\nu \right)}\right)$$

The derived equations prove that the sensor’s output voltage depends solely on the Poisson’s ratio ν and total stressed cross-sectional area A of the substructure when the y-axis pressure is constant. For a tunable Poisson’s ratio substructure with predetermined y-axis pressure, ν exclusively depends on the configuration and design parameters of the substructure. Consequently, systematic investigation of the pressure–Poisson’s ratio relationship can effectively enhance the linearity and other performance metrics of magnetostrictive pressure sensors [18].

To achieve an optimal linear relationship between the sensor’s output voltage and applied pressure, the following objective optimization equation is utilized to analyze the linear relationship between output voltage and pressure:

(22) $$\overset{\cdot }{U_{2}}=C_{1}F_{i}+C_{2}$$

where $C_{1}$ and $C_{2}$ are undetermined coefficients, and $F_{i}$ represents the ideal force function of the sensor.

With the design parameters of the substructure fixed, and considering the variation in Poisson’s ratio with strain or external force under large deformation, the theoretical formula for sensor output voltage is simplified as follows:

(23) $$\overset{\cdot }{U_{2}}=C_{3}\left(1+\frac{C_{4}}{C_{5}+C_{6}F_{y}\left(1-2\nu \right)}\right)$$

where the constant terms are respectively:

(24) $$\left\{\begin{array}{l} C_{3}=j\omega \overset{\cdot }{I_{1}}\frac{\mu_{0}^{2}nS_{2}}{\mu_{1}}\\ C_{4}=M_{s}^{2}A\\ C_{5}=2K_{u}A\\ C_{6}=3\lambda_{s} \end{array}\right.$$

Simultaneous Equations (19) and (20) are obtained as follows:

(25) $$F_{i}=\frac{1}{C_{1}}\left(C_{3}-C_{2}+\frac{C_{3}C_{4}}{C_{5}+C_{6}F_{y}\left(1-2\nu \right)}\right)$$

For a tunable Poisson’s ratio substructure, the Poisson’s ratio can be expressed as a function of the strain or external pressure. By adjusting the Poisson’s ratio function, the actual force of the sensing material can be aligned with the ideal force function.

Therefore, assuming that $F_{y}=F_{i}$, the expression for tunable Poisson’s ratio ensuring that the force on the sensing material meets the requirements is derived as:

(26) $$\nu =\frac{1}{2}+\frac{1}{2C_{6}F_{y}}\left(C_{5}-\frac{C_{3}C_{4}}{C_{1}F_{y}+C_{2}-C_{3}}\right)$$

Following the acquisition of the aforementioned theoretical analysis results, key determining parameters for the mechanical properties of the substructure and the linearity of the sensor were extracted and integrated. The theoretical results completed the preparation for subsequent finite element simulations and experimental design.

### 2.4. Finite Element Method Analysis

**Figure 4:**
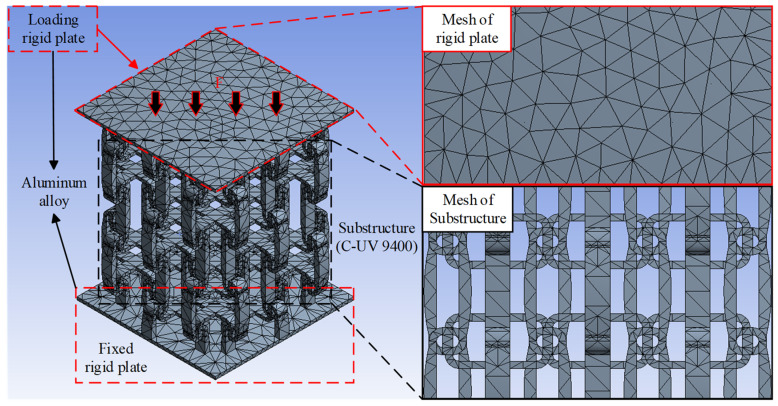
Finite element model of tunable Poisson’s ratio substructure.

In ANSYS Workbench 16.0 software, the substructure material is defined as C-UV 9400 photosensitive resin, with its physical properties specified as follows: a density of 1150 kg/m^3^, a flexural strength of 73 MPa, a tensile strength of 56 MPa, a Young’s modulus of 2678 MPa, and a Poisson’s ratio of 0.44. For the loading and fixed-end rigid plates, an aluminum alloy was selected, characterized by a density of 2770 kg/m^3^, a tensile strength of 310 MPa, a yield strength of 280 MPa, a Young’s modulus of 7.1 GPa, and a Poisson’s ratio of 0.33. To achieve enhanced simulation accuracy, tetrahedral elements of varying densities were employed, generating the mesh shown in Figure 4.

The generated mesh consists of 84,499 nodes and 38,441 elements. The top and bottom surfaces were rigidly connected to the loading plates, with zero relative displacement. A displacement was applied to the top starting from 0 mm, at a rate of 1 to 2 mm/s, with a maximum displacement of 15 mm. The analysis settings incorporated large deformation effects; such a setting makes the mechanical model of FEM different from the theoretical calculation model. The large deformation mode considers nonlinear deformation of the material, which will gradually change with the increase in strain. For the substructure of this paper, displacement of components during large deformations will deviate in direction due to rotation, which reduces the Poisson’s ratio of the substructure insignificantly. The above differences have a negligible impact on the research, so the FEM model chose the large deformation assumption that is closer to the actual situation.

**Figure 5:**
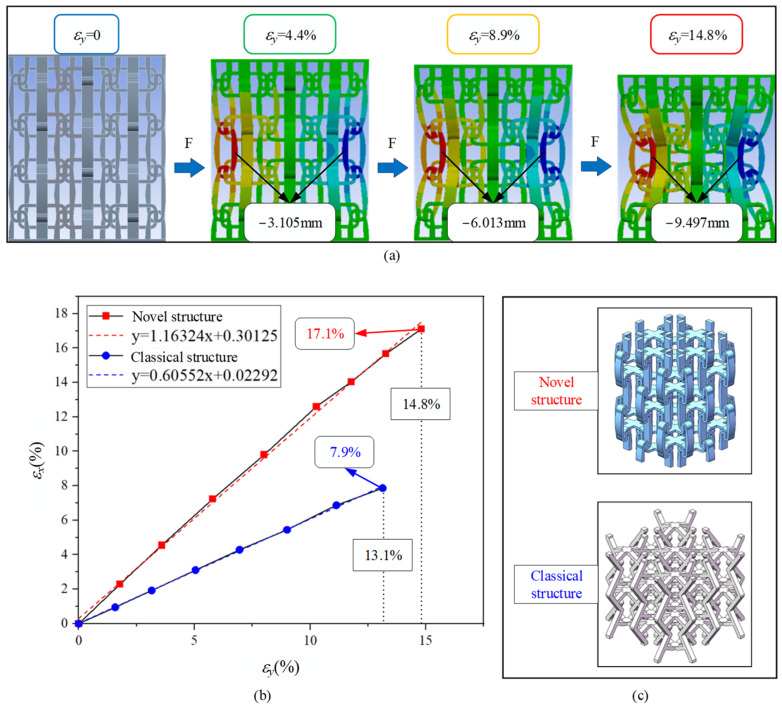
(**a**) Deformation simulation results. (**b**) Simulation result of strain linearity. (**c**) Comparison of two substructure models.

The FEM results are shown in Figure 5. As the compressive strain increased from 0 to 14.8%, the central region of the substructure exhibited a uniformly increasing transverse compressive deformation, reaching a maximum value of −9.497 mm. In the TPR substructure, the coefficient of determination for the linear regression between the transverse and axial strains is R^2^ = 0.99816, which is comparable to the R^2^ = 0.99935 of the classical structure. This indicates that the proposed structure retains the characteristic of highly uniform strain inherent in tetra-chiral honeycomb structures. Notably, the maximum transverse strain increased to 17.1%, significantly enhancing the deformation performance of the tetra-chiral honeycomb. Consequently, an average compressive Poisson’s ratio (the negative slope of the regression line) of −1.16324 was achieved, which is approximately double that of the conventional structure. Moreover, the Poisson’s ratio obtained by FEM is slightly lower than the theoretical value of −1.27039, which is consistent with the use of an ideal elastic model in theoretical calculations and the assumption of large deformation in FEM settings in the study.

### 2.5. Experiment Setup

**Figure 6:**
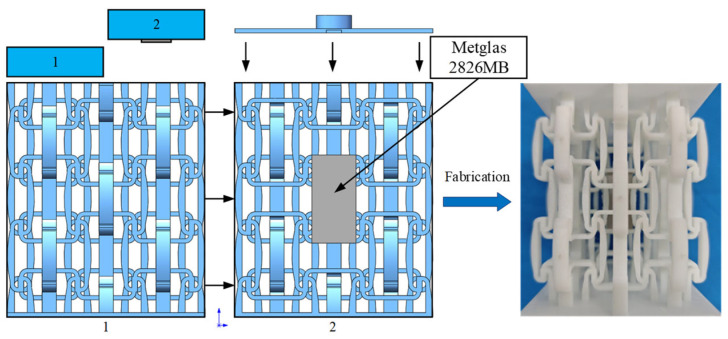
The model and the actual sample of the sensor.

The aforementioned TPR substructure was fabricated using a photopolymerizable resin material (C-UV 9400, Shanghai, China), while the 2826MB sensing element was bonded to the substructure using a modified acrylate adhesive (AILIKE-A8, Shenzhen, China). The final sensor sample obtained is shown in Figure 6.

**Figure 7:**
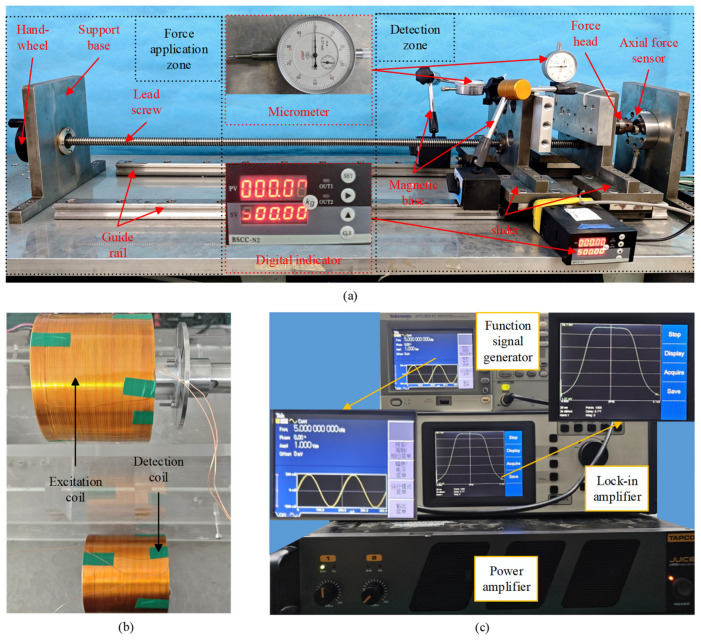
(**a**) The experimental platform. (**b**) The experimental coils. (**c**) The experimental apparatus.

The experimental setup used in this study, including the experimental platform and experimental apparatus, is illustrated in Figure 7.

In the bias module, a function generator coupled with a power amplifier supplies current to the excitation coil, generating a bias magnetic field that maintains the magnetostrictive material in a magnetized state. The loading module incorporates a tunable handwheel mechanism that precisely positions the clamping plates for both the alignment and application of desired pressure. A precision indenter mounted on one clamping plate contacts a force sensor fixed at the terminal end of the test bench, enabling accurate measurement of the applied load. Within the detection module, the sensing coil interfaces with an oscilloscope for real-time voltage monitoring and data acquisition of the sensor’s output signal.

During the experiment, the sensor was securely mounted in the loading zone, and the force applied was measured using the load cell contacted by the precision indenter. The handwheel was adjusted to drive the clamping plates along the lead screw and linear guide rails, applying controlled pressure to the sensor. Multiple sets of force readings and corresponding output voltage readings were recorded.

The acquired data were then plotted to analyze the pressure–output voltage relationship under various loading conditions. Linear regression was performed to evaluate the sensor’s linearity performance, with residual error analysis providing quantitative assessment metrics.

## 3. Results and Discussion

### 3.1. Strain Results and Discussion

**Figure 8:**
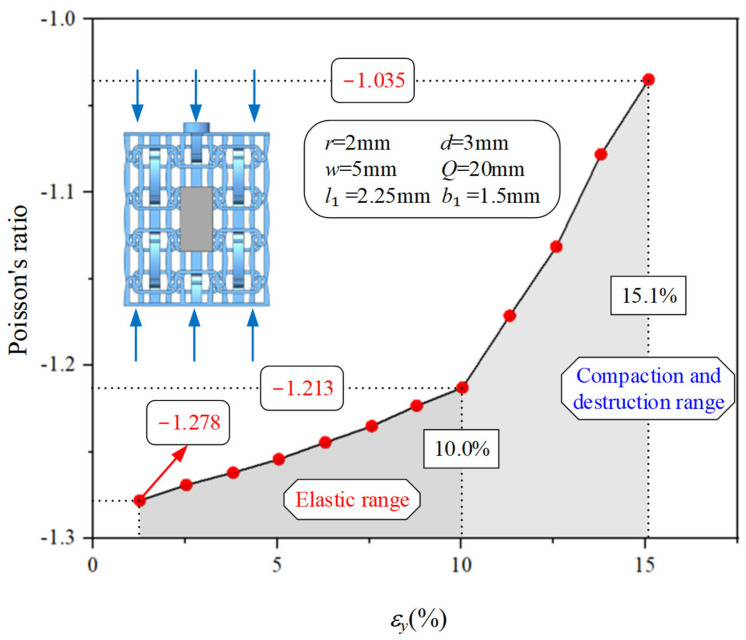
Results for the Poisson’s ratio performance of the substructure.

Figure 8 presents the experimental results for the Poisson’s ratio performance of the substructure. The Poisson’s ratio curve during the deformation of the substructure can be divided into two distinct stages: the elastic stage and the densification and failure stage. At the onset of loading, the substructure exhibits a minimum Poisson’s ratio of −1.278. Within the elastic stage, characterized by small *y*-axis strains, the Poisson’s ratio remains relatively stable, with its value gradually decreasing from −1.278 to −1.213. This indicates that the substructure maintains a smooth and stable trend in Poisson’s ratio within the elastic deformation range, reflecting a uniform elastic response. Once the strain reaches 10.0%, the Poisson’s ratio begins to rise rapidly with further increases in axial strain, signaling the entry of the substructure into the densification and failure stage. During this phase, the primary load-bearing components, such as the internal circular arc members, begin to fail. This is accompanied by a densification process caused by the closure of local pores, both of which collectively lead to a significant degradation of the negative Poisson’s ratio (NPR) effect.

**Figure 9:**
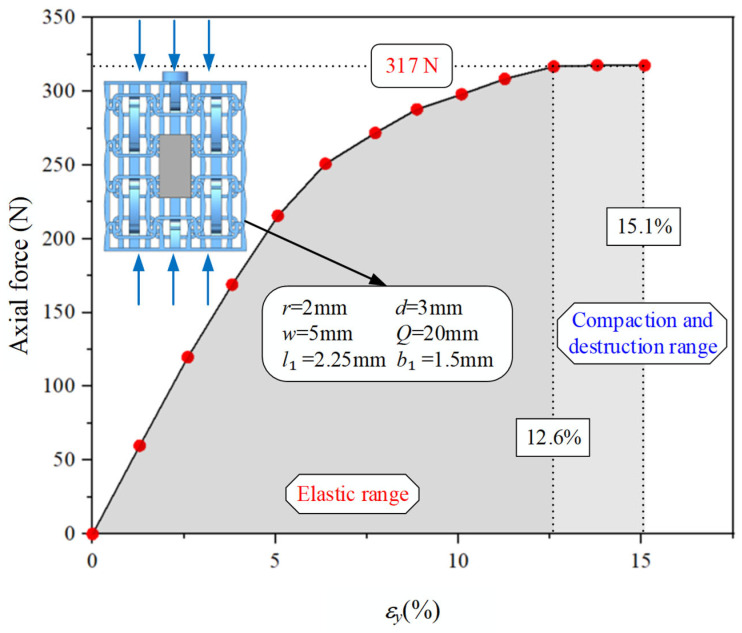
Results for the load-bearing capacity of the substructure.

The load-bearing capacity of the substructure was experimentally evaluated, as shown in Figure 9. The load-bearing curve of the substructure similarly exhibits two primary stages: the elastic stage and the densification and failure stage. Unlike the previous results, the load-bearing capacity displays a characteristic convergence toward an extreme value, entering a complete plateau region during the densification and failure process. The maximum load-bearing capacity before failure is 317 N at a strain of 12.6%. Throughout the elastic deformation stage, the load-bearing curve exhibits a convex profile, which is attributed to the large-deformation mode of the actual structure. Subsequently, during the densification and failure stage, the growth rate of the load-bearing capacity significantly decelerates due to the failure of components such as circular arc hinges, eventually reaching a state where the load no longer increases.

**Figure 10:**
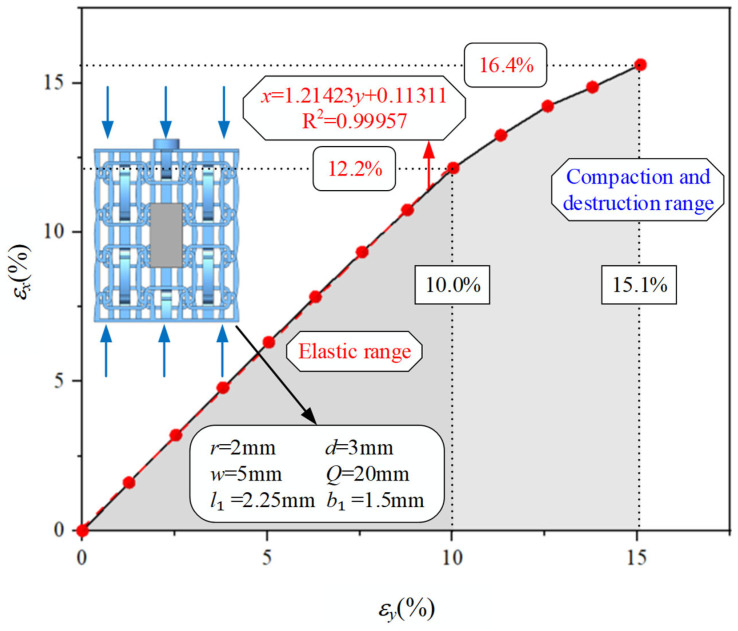
Results for the strain linearity of the substructure.

Finally, considering the influence of the substructure performance on the sensor’s output characteristics, the strain linearity of the substructure was investigated, as shown in Figure 10. Within the elastic limit, the linear fitting relationship between the y-axis and x-axis strains of the substructure is given by *x* = 1.21423*y* + 0.11311 (R^2^ = 0.99957), indicating a robust linear correlation between the generated axial and transverse strains. Consistent with the results of the Poisson’s ratio experiment, the strain linearity of the substructure deteriorates significantly after the strain reaches 10.0% due to local component failure and overall structural densification.

In the elastic stage, the high absolute value of the average Poisson’s ratio ($\left|\nu \right|$ = 1.21423) and the high-reliability linear correlation (R^2^ = 0.99957) strongly validate the elastic hypothesis of the substructure within this range. Furthermore, the experimental data exhibit a high degree of convergence with the analytical results from both theoretical calculations and finite element method (FEM) simulations, demonstrating the application potential of the tetra-chiral TPR substructure. These experimental findings provide crucial evidence supporting the proposed method of utilizing a TPR substructure as a substructure to enhance the performance of magnetostrictive sensors.

The comparison of mechanical properties obtained by different research methods is shown in Table 1.

### 3.2. Electrical Results and Discussion

Fundamental performance calibration experiments are a primary requirement for enhancing sensing performance and enabling the practical application of sensors. Since this study focuses on two key performance metrics, sensitivity and linearity, the subsequent calibration experiments are oriented toward analyzing their performance.

Sensitivity is defined as the change in the sensor’s output resulting from a unit change in the input quantity. For the inverse-effect magnetostrictive strain sensor discussed in this paper, the strain sensitivity can be expressed as:

(27) $$S_{a}=\frac{\overset{\cdot }{\Delta U_{2}}}{\Delta \varepsilon_{y}}$$

Linearity, on the other hand, can be determined through the stability of the output curve’s slope (i.e., the stability of the sensitivity) and the coefficient of determination R^2^ from linear regression analysis. Consequently, the calibration of the sensor allows for the acquisition of these two primary performance metrics.

**Figure 11:**
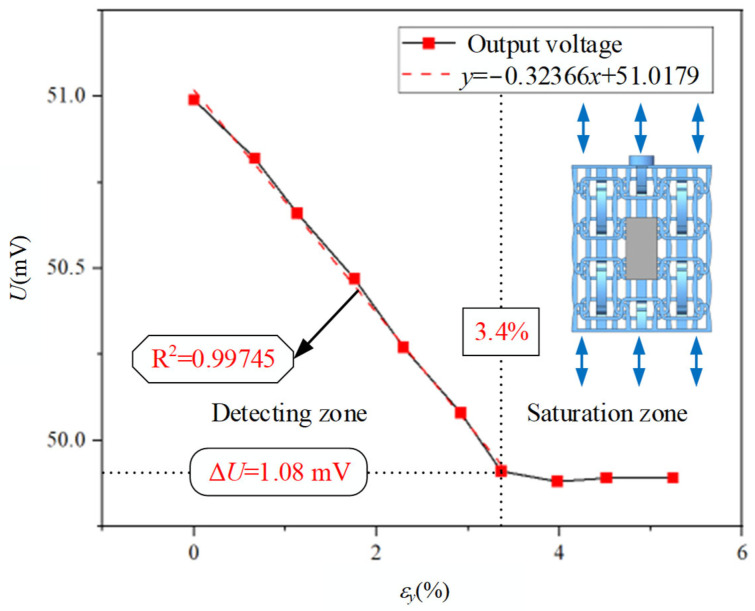
Results for the calibration experiment of the sensor.

Figure 11 illustrates the output voltage results from the sensor calibration experiment. On either side of the 3.4% strain threshold, the sensor’s output voltage can be divided into a detection zone and a saturation zone. Within the detection zone, the output voltage decreases steadily, reaching a maximum effective voltage of 1.08 mV at the 3.4% strain point. The linear fit between the output voltage and strain within this detection zone is expressed as: *y* = −0.32366*x* + 51.0179. Moreover, the coefficient of determination R^2^ for this linear relationship reaches 0.99745, which fundamentally demonstrates that the sensor developed in this study possesses high sensitivity and excellent linear output performance.

**Figure 12:**
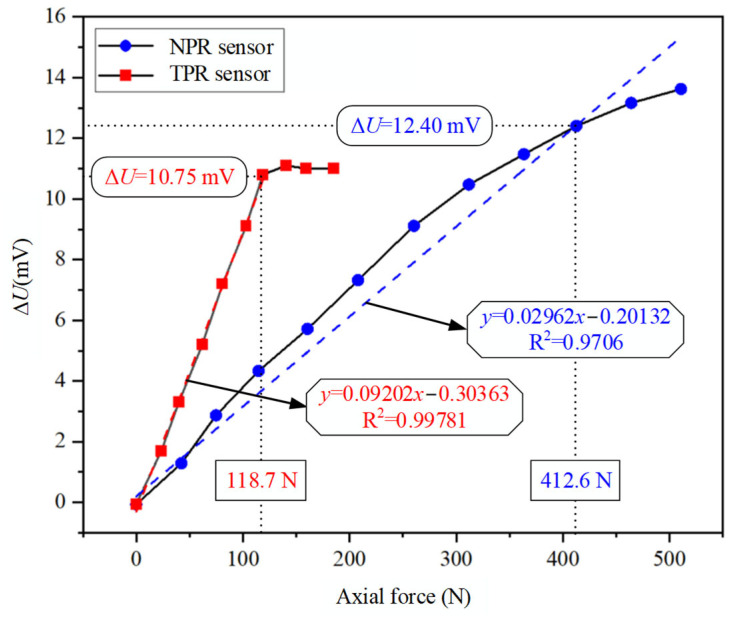
Results for the linearity experiment of the sensor.

Based on the calibration results, the sensor primarily possesses the sensing region and the saturation region. Figure 12 illustrates the output voltage from the sensor linearity experiment. Within the sensing region, the sensor demonstrates a highly linear relationship between the applied axial force and the output voltage, with a coefficient of determination of R^2^ = 0.99781, indicating a robust linear regression fit. Concurrently, the maximum detectable force within the sensing region occurs at 118.7 N, reaching an output of 10.75 mV, which further validates the superior sensitivity of the sensor. When the axial force exceeds 118.7 N, the sensor enters the magnetic saturation region, where the output signal remains essentially constant. Furthermore, by comparing these results with the output voltage data of another sensor utilizing a negative Poisson’s ratio substructure [18], the sensor proposed in this study possesses significantly enhanced sensitivity and linearity, while there is no overtravel zone demonstrated.

The hysteresis error of a sensor is defined as the relative error between its output during the forward and reverse strokes. Specifically, it is the ratio of the maximum deviation between the output signal curves to the sensor’s full-scale output voltage:

(28) $$\gamma_{H}=\frac{\Delta H_{m}}{V}$$

where $\Delta H_{m}$ is the maximum deviation between the output signal curves and V is the full-scale output voltage.

Hysteresis error is a direct reflection of the stability of the sensor’s output signal. A low hysteresis error demonstrates superior consistency between forward and reverse stroke outputs, indicating that the sensor’s output characteristics are minimally affected by the existing loads applied within its measurement range.

**Figure 13:**
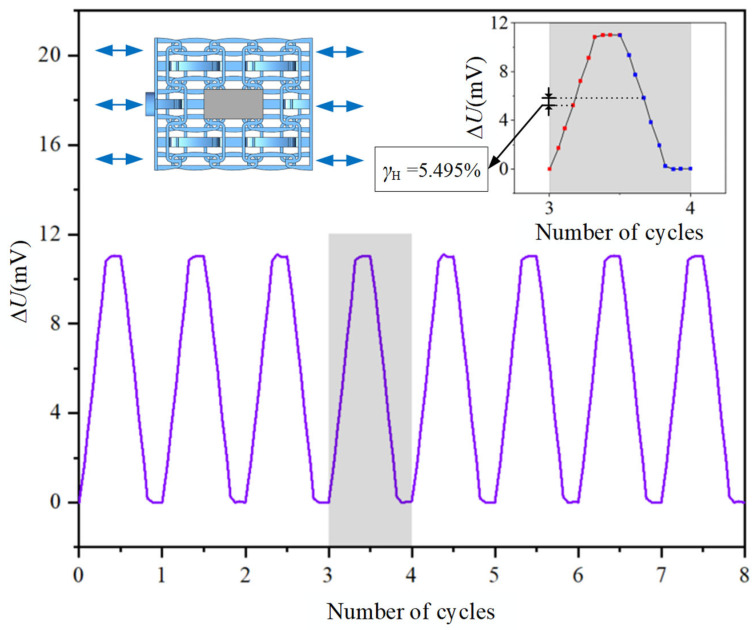
Results for the hysteresis characteristics of the sensor. The shaded area represents the cycle with the maximum error in all cycles, while the red and blue dots represent the rising and falling stages of the signal, respectively.

The results of the sensor’s hysteresis characteristic experiments are shown in Figure 13. Eight cycles of loading–unloading experiments were conducted to evaluate the hysteresis error of the sensor. Throughout the eight cycles, the output signals maintained a highly symmetrical and corresponding relationship between the forward and reverse strokes. The maximum hysteresis error, occurring during the fourth cycle, was only 5.495%, which confirms the excellent hysteresis characteristics of the sensor. Furthermore, the signals across different cycles were closely aligned, indicating that the sensor also exhibits strong drift performance.

### 3.3. Application Experiment Results and Discussion

**Figure 14:**
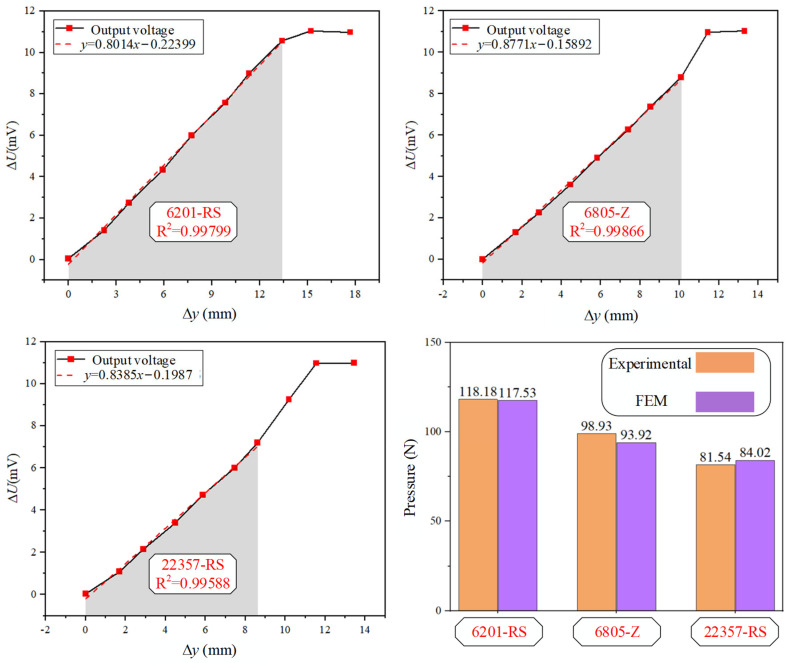
Results of the press-fitting forces for different bearings.

The experimental results for the press-fit force detection of various bearings are shown in Figure 14. For the 6201-RS bearing, the linear regression equation of the output voltage is *y* = 0.8014*x* − 0.22399, with a coefficient of determination R^2^ of 0.99799, and the calculated maximum press-fit force is 118.18 N. For the 6805-Z bearing, the linear relationship equation is *y* = 0.8771*x* − 0.15892, with an R^2^ of 0.99866, and a maximum press-fit force of 98.93 N. Finally, for the 22357-RS bearing, the linear regression equation is *y* = 0.8385*x* − 0.1987, with an R^2^ of 0.99588, and a maximum press-fit force of 81.54 N.

The data from each experimental group demonstrate a highly linear signal output throughout the press-fit stroke, validating the enhanced linearity performance of the sensor. Furthermore, a comparison between the experimental peak pressure results and the finite element method (FEM) simulation results for bearing fit pressure shows that the detected peak forces for all three bearing types are highly consistent with the FEM results. This proves the sensor’s capability for high-precision axial force detection.

**Figure 15:**
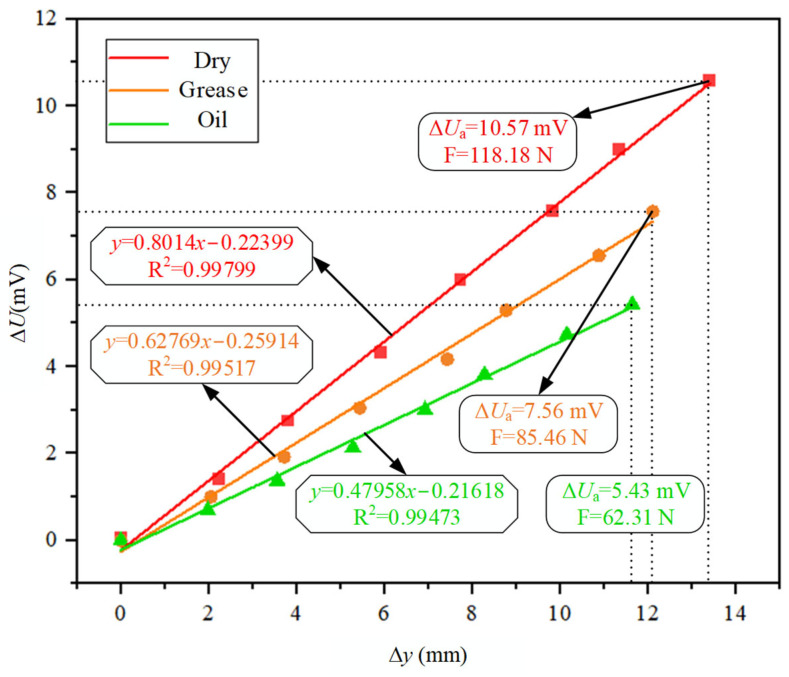
Results of bearing lubrication press-fit force.

The experimental results for the various lubrication groups are shown in Figure 15, and the bearing selected is the standard bearing 6201-RS used in the previous section.

In the dry (unlubricated) group, the maximum voltage change for the press-fit force detection was 10.57 mV, corresponding to a detected force of 118.18 N. The grease-lubricated group exhibited a maximum voltage change of 7.56 mV, corresponding to a force of 85.46 N. The oil-lubricated group showed the lowest maximum voltage change at 5.43 mV, corresponding to a force value of 62.31 N.

The sensor remains capable of capturing effective measurement data for the smaller press-fit forces generated during assembly under lubricated conditions, demonstrating its excellent sensitivity performance. Furthermore, the detected voltages across all three experimental groups maintain a highly linear characteristic, with their respective coefficients of determination R^2^ reaching 0.99799, 0.99517, and 0.99473. This indicates that the sensor developed in this study maintains a linear signal output even under the influence of smaller axial forces, proving its robust linearity performance.

**Figure 16:**
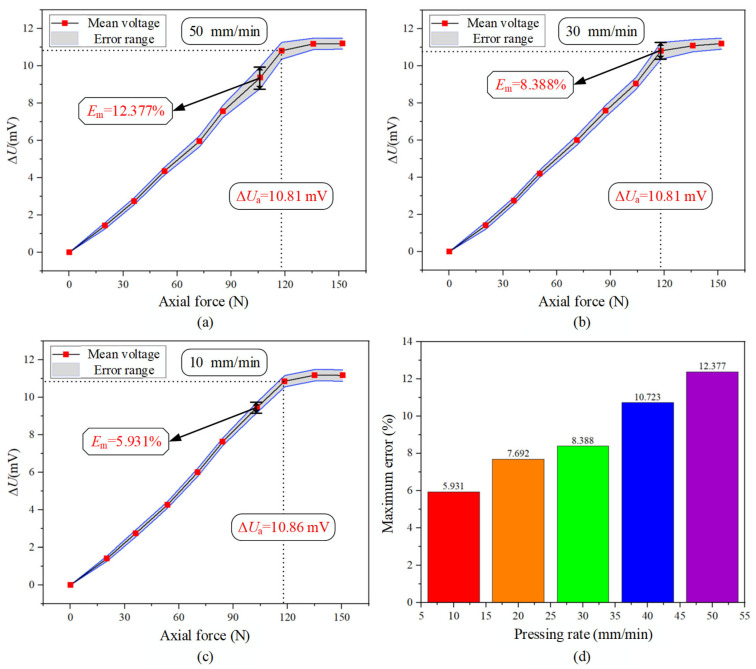
(**a**) Results under 50 mm/min. (**b**) Results under 30 mm/min. (**c**) Results under 10 mm/min. (**d**) Comparison of the maximum voltage error across all rates.

Finally, to verify the repeatability and stability of the sensor data, bearing press-fit force detection experiments were conducted at different assembly insertion rates. Five groups of press-fit rates were tested within the range of 10 to 50 mm/min, increasing by 10 mm/min per group, with five repeated loading tests performed within each group. The voltage error intervals (shaded areas) and average output voltages for three representative rates, 50 mm/min, 30 mm/min, and 10 mm/min, are shown in Figure 16.

The average maximum voltage changes for the three groups were 10.81 mV under 50 mm/min, 10.81 mV under 30 mm/min, and 10.86 mV under 10 mm/min. These values are highly consistent, indicating the excellent operational stability of the sensor. Furthermore, the maximum output voltage error of the sensor was 12.377% under 50 mm/min and 8.388% under 30 mm/min. At the 10 mm/min rate, the sensor error reached a minimum value of 5.931%. Overall, the sensor exhibits small dynamic errors, demonstrating its practical application value. The sensor’s dynamic error shows a positive correlation with the press-fit rate when comparing the error across all groups.

Through a series of bearing press-fit force experiments, this section further substantiates the advantages of the developed sensor in terms of high linearity and high sensitivity, while validating the stability of its signal. The results demonstrate that the sensor designed in this study possesses sufficient potential for practical application, providing a final realization of the value of the experiments and the overall study.

## 4. Considerations and Perspectives

This study presented a magnetostrictive pressure sensor employing a tunable Poisson’s ratio (TPR) chiral honeycomb substructure, with the objective of enhancing sensor linearity. The innovation lies in engineering the substructure’s Poisson’s ratio profile to linearize the strain of the Metglas2826MB sensing material, thereby addressing the nonlinear output characteristics commonly observed in substructure-optimized magnetostrictive sensors.

The FEM-predicted average compressive Poisson’s ratio of ν = −1.1632 and transverse-to-axial strain linearity of R^2^ = 0.99816 of the structure were corroborated by the experimentally measured Poisson’s ratio of ν = −1.21423 with R^2^ = 0.99957 over the elastic deformation range. Both simulation and experiment identified two distinct deformation stages: an elastic range where the Poisson’s ratio remained within −1.278 to −1.213, followed by a densification-and-failure stage beyond 10% strain where local component failure degraded the strain. This consistency validates the theoretical model derived from the flexibility matrix and confirms that the bending of hinge components dominates the deformation behavior as assumed. The absolute value and stability of the Poisson’s ratio of the substructure in our research are significantly better than the performance of most tetra-chiral NPR structures in existing research [29,30].

Experimentally, the sensor achieved a calibration linearity of R^2^ = 0.99745 and a continuous linear force response up to 118.7 N with a maximum output voltage variation of 10.75 mV, with a well-defined detection zone extending to 3.4% strain followed by magnetic saturation. Comparison with a conventional negative Poisson’s ratio substructure sensor [18] confirmed that the proposed TPR design simultaneously enhanced both sensitivity and linearity while eliminating the overtravel zone. Moreover, the maximum load-bearing capacity of the TPR substructure reached 317 N at 12.6% strain, well above the 118.7 N sensing limit, and the hysteresis error remained within 5.495% over eight loading cycles. Such experimental results have verified comprehensive excellent performance of our sensor.

The multi-type bearing press-fit experiments (6201-RS, 6805-Z, and 22357-RS) confirmed the sensor’s robust linearity across different assembly configurations, with R^2^ values of 0.99799, 0.99866, and 0.99588, respectively. The detected peak press-fit forces (118.18 N, 98.93 N, and 81.54 N) showed strong agreement with FEM simulation results for bearing fit pressure, validating the sensor’s quantitative accuracy. Under lubricated conditions, the sensor maintained high linearity (R^2^ of 0.99799 for dry, 0.99517 for grease-lubricated, and 0.99473 for oil-lubricated assembly) despite progressively reduced axial forces (118.18 N, 85.46 N, and 62.31 N, respectively), demonstrating operational robustness. Such experimental results verify the linearity and other key performance indicators of the sensor, and demonstrate the sensor’s value in applications such as bearing assembly.

It is noteworthy that the AILIKE-A8 modified acrylate adhesive used to bond the Metglas2826MB sensing element to the substructure introduces a compliant interface not captured in the idealized FEM and theoretical assumptions. Experimental results nonetheless demonstrated an excellent strain transfer efficiency, as evidenced by the high calibration linearity of R^2^ = 0.99745 and the sensor’s ability to detect small force variations under lubricated press-fit conditions (output voltage changes of 5.43 mV for oil-lubricated assembly, with R^2^ = 0.99473).

The bearing press-fit rate experiments revealed a positive correlation between dynamic output error and insertion rate, with errors increasing from 5.931% at 10 mm/min to 12.377% at 50 mm/min. Nevertheless, in the field of bearing assembly, the industrial tolerance of the dynamic fluctuation error range for press-fit force is generally 15%, which demonstrates that the dynamic error of our sensor meets the tolerance.

However, the minor sensitivity variation across bearing types suggests that application-specific calibration may be necessary for the sensor. Moreover, the current manual loading mechanism introduces rate-dependent variability that could be mitigated through automated actuation. Lastly, further reduction in the dynamic error at higher operational speeds remains a target for optimization.

As a prospect, future studies incorporating rate-dependent constitutive models would enable prediction of the sensor’s dynamic performance envelope. Additionally, temperature fluctuations, known to affect both the elastic modulus of polymer resins and the magnetoelastic coefficient of Metglas2826MB [3], should be considered for applications beyond the ambient temperature under controlled laboratory environments. Finally, research on utilizing TPR substructures with uniform and linear strain in other sensors such as piezoelectric sensors will also become a possible direction.

A comparison of the proposed sensor with previous studies is shown in Table 2.

## 5. Conclusions

Experimental characterization of the TPR substructure revealed three noteworthy mechanical properties. First, the substructure exhibited a stable negative Poisson’s ratio ranging from −1.278 to −1.213 within the elastic deformation regime, confirming sustained auxetic behavior during compressive loading. Second, the substructure achieved a maximum load-bearing capacity of 317 N at 12.6% strain, providing adequate structural strength for sensor applications. Third, a highly linear relationship between axial and transverse strains was established (x = 1.21423y + 0.11311), providing the mechanical foundation for the sensor’s electrical linearity. The experimental results demonstrated close agreement with both theoretical predictions and finite element simulations.

The integrated magnetostrictive sensor exhibited excellent electrical performance. Calibration experiments revealed a linear detection zone with R^2^ = 0.99745, confirming the sensor’s high linearity. Force linearity tests demonstrated a continuous linear response up to 118.7 N with a maximum output of 10.75 mV, with no overtravel zone observed. Hysteresis characterization over eight loading–unloading cycles revealed a maximum hysteresis error of only 5.495%, with closely aligned signals across cycles indicating strong drift stability. Compared with a sensor employing a conventional negative Poisson’s ratio substructure, the proposed sensor achieved significantly enhanced sensitivity and linearity.

The practical utility of the sensor was demonstrated through bearing press-fit force monitoring experiments. Multi-type bearing tests (6201-RS, 22357-RS, and 6805-Z) yielded high linearity across all configurations, with R^2^ values exceeding 0.995, and detected peak press-fit forces showed strong agreement with FEM simulation results. Lubrication condition experiments revealed that the sensor maintained reliable linear output under dry assembly of R^2^ = 0.998, grease-lubricated of R^2^ = 0.995, and oil-lubricated of R^2^ = 0.995. Press-fit rate experiments across 10–50 mm/min showed consistent average maximum voltage output ranging from 10.81 to 10.86 mV, with dynamic error ranging from 5.931% at 10 mm/min to 12.377% at 50 mm/min. These results collectively validate the sensor’s capability for in situ press-fit quality monitoring in rotating machinery assembly.

Several limitations warrant acknowledgment. First, the sensor’s sensitivity exhibited minor variation across bearing types, suggesting that type-specific calibration may be necessary. Second, the manual loading mechanism introduces rate-dependent variability. Third, the dynamic error at higher press-fit rates (12.38% at 50 mm/min) indicates that the current sensor configuration may require optimization for high-speed assembly. Future work will focus on extending the force range through parametric optimization, integrating wireless signal transmission for fully passive operation, and investigating the sensor’s dynamic response under impact loading conditions.

## Figures and Tables

**Table 1:** Comparison of the mechanical properties from different research methods.

Items	Theoretical	FEM Simulation	Experimental
Poisson’s ratio	−1.27039	−1.16324	−1.21423
Strain linearity	N/A	R^2^ = 0.99816	R^2^ = 0.99957

**Table 2:** Comparison of the proposed sensor with previous literature.

Items	Proposed Sensor	The Sensor in [22]	The Sensor in [18]
Linearity	R^2^ = 0.99781	R^2^ = 0.988	R^2^ = 0.9706 (Including overtravel)
Sensitivity	0.09202	0.0021 (Detecting Fe-Mn alloy)	0.02962 (Including overtravel)
Stability	5.931%	3.59%	Not considered

## Data Availability

The data presented in this study are available on request from the corresponding author.
